# AI-driven analysis establishes the single base substitution signatures as personalized prognostic predictors for five-year survival of gastric cancer

**DOI:** 10.1016/j.gendis.2023.05.021

**Published:** 2023-07-13

**Authors:** Zhenzhang Li, Lingqing Xu, Wen Luo, Shaoan Zhang, Chunyu Hou, Xiaohong Xu, Xubei Peng, E. Shiju, Janak L. Pathak, Shizhen Zhang, Jiawei Liu, Linhai Li, Yang Li

**Affiliations:** a School of Biomedical Engineering, The Sixth Affiliated Hospital, Guangzhou Medical University, Guangzhou, Guangdong 511436, China; b College of Mathematics and Systems Science, Guangdong Polytechnic Normal University, Guangzhou, Guangdong 510665, China; c The Second Affiliated Hospital, Guangzhou Medical University, Guangzhou, Guangdong 510260, China; d Center for Learning Sciences and Technologies, The Chinese University of Hong Kong, Shatin, New Territories 999077, China; e Affiliated Stomatology Hospital of Guangzhou Medical University, Guangzhou Medical University, Guangzhou, Guangdong 510182, China; f International School of Photonics, Cochin University of Science and Technology, Kochi, Kerala 682022, India

AI-driven genetic engineering, as a burgeoning diagnostic tool, can offer predictive information on the five-year survival rate (FYSR) in the setup of a prognostic therapeutic schedule. This approach provides the individuality and accuracy of prognosis for FYSR of gastric cancer (GC). Unlike traditional neoplasm staging criteria, our technique ensures accuracy and individuality without relying on statistical data and empirical study. Here, we designed a gene mutation analysis algorithm (cumulative contribution abundance, CCA, Supplementary Material 1.1) to drive a single base substitution (SBS) signature to score GC prognosis because the algorithm can better represent the relationship between genes and mutational signatures. We found a new prognostic survival factor (SBS44) of GC and verified that SBS18 can also be utilized in this capacity. Then, a GC FYSR predictive AI model was constructed that combined the SBS44 and SBS18 (SBS44&18) signatures as characteristic variables and obtained high accuracy (AUC: 0.9194, 95% CI: 0.8357–1). Our results suggest that this technique is beneficial for accurate prognostic assessment and provide a new idea for clinical stratified treatment.

First, the somatic mutation profiles of 462 GC patients were pooled, which were taken from previous genomic studies (Table S1, 2; Supplementary Material 2.2). Information on a total of 11, 570, 459 mutations was collected, including 9,016,437 single-base mutations and 2,554,022 insertion and deletion mutations. For those GC samples, approximately 7% (31/462) were hypermutated (Fig. S1). They were mostly found in the MSI status (microsatellite instability), which was represented in TCGA's MSI subtype, and the intestinal type and survival status (Fig. A2 of Supplementary Material 3.2).

## SBS44∗ and SBS18∗ as important mutational signatures in GC

**Figure 1:**
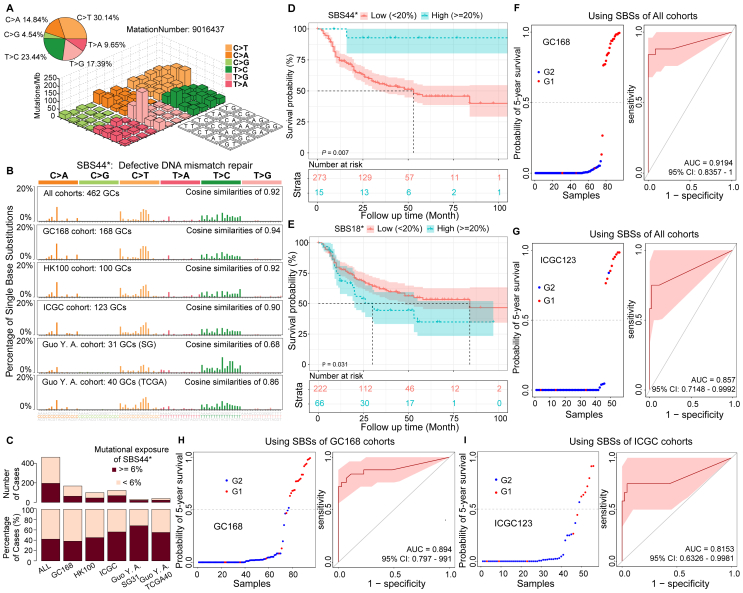
SBS44∗ and prognosis of SBSs in gastric cancer. **(A)** Lego plot representation of mutation patterns in 462 GC cases. The inset pie chart shows the proportion of six categories of mutation patterns. **(B)** SBS44∗ is depicted using a 96-substitution classification defined by the substitution type and sequence context immediately 5′ and 3′ to the mutated base in different datasets. The cosine similarity between SBS44∗ of each dataset and COSMIC SBS44 is calculated. **(C)** Distribution of cases with SBS44∗ activity (top) and percentage of cases with SBS44∗ activity in the different datasets (bottom). **(D)** Kaplan–Meier survival analysis stratified by mutational exposure of SBS44∗ status. **(E)** Kaplan–Meier survival analysis stratified by mutational exposure of SBS18∗ status. SBSs classify five-year survival in gastric cancer. **(F–I)** Classification of the test set consisted of G2 (blue) and G1 (red) from the GC168 and ICGC123 cohorts, respectively. The receiver operating curves (ROCs) for those datasets are provided.

To further understand the mutational process in GC, we delineated mutational signatures from the genome mutation map. The predominant mutations were C > T (30.14%) and T > C (23.44%) when we first tallied the 96 single nucleotide variations that may exist in the trinucleotide context in each GC sample (Fig. 1A). Subsequently, using the COSMIC^1^ feature nomenclature, we categorized 10 mutational signatures from the 462 GC genome data that had various levels of mutagenic activity (Fig. S2, 3). These 10 signatures exhibited high cosine similarity to the COSMIC mutation signatures SBS18, SBS2, SBS17a, SBS28, SBS1, SBS17b, SBS58, SBS52, SBS3, and SBS44 on COSMIC (cosine coefficients were 0.97 for SBS18∗, 0.83 for SBS2∗, 0.94 for SBS17a∗, 0.92 for SBS28∗, 0.98 for SBS1∗, 0.97 for SBS17b∗, 0.91 for SBS58∗, 0.82 for SBS52∗, 0.82 for SBS3∗, and 0.92 for SBS44∗; Fig. S3, asterisks indicate the signatures analyzed by *RNMF*^2^ analysis in this work). This shows that these characteristics are important signatures of GC, such as SBS18∗, which is in accordance with previous findings.

To confirm these potentially important signatures in GC, we performed independent feature extraction analysis on each dataset and found that some signatures were also present in these independent cohorts, such as SBS44∗ (cosine coefficient was 0.94 of GC168 cohort with 168 cases, 0.92 of HK100 cohort with 100 cases, 0.9 of ICGC cohort with 123 cases, 0.68 of Guo Y.A. cohort with 31 cases, 0.86 of Guo Y.A. cohort with 40 cases; Fig. 1B), which enriched a large number of C > T and T > C type mutations and was linked to the deficiency in DNA mismatch repair. Then, we conducted an SBS44∗ scan throughout the entire genome of these tumors and discovered that at least one-third of the samples contributed more than 6% to this signature in each dataset (Fig. 1C). Interestingly, this signature was very active in hypermutated samples (Fig. A2 of Supplementary Material 3.2, 83.87% *vs*. 39.21% of cases, *P* < 0.00001, Fisher's test with "two-sided"). Linear correlation analysis revealed that the number of mutations in the sample was strongly related to the number of mutations that contributed to SBS44∗ (Spearman *R* = 0.73, *P* < 0.001), as well as the mutation number of samples and the mutational exposure of samples to SBS44∗ (Spearman *R* = 0.001) 0.19, *P* < 0.001; Fig. S4A).

## SBS44∗ and SBS18∗ as prognostic factors for survival in gastric cancer

To explore the association of these mutational signatures with prognosis, we performed a Kaplan–Meier survival analysis. Interestingly, we found that in tumors with mutational exposure of SBS44∗, more than 20% was significantly associated with a better survival outcome in this cohort (Fig. 1D). In contrast, those cases with more than 20% mutational exposure of SBS18∗ showed a significantly poor prognosis (Fig. 1E), which has been previously reported.^3^ Unfortunately, the SBS17b∗ feature is not a prognostic factor for survival in GC (Fig. S5), which also verifies the above assumptions.

## The prediction model of FYSR for gastric cancer

To further investigate the relationship between genes and two (SBS18∗ and SBS44∗) prognosis-related mutational signatures for prognosis evaluation, we used a random forest algorithm^4^ in machine learning to screen prognostic targets for GC based on the CCA model.

According to the follow-up time and survival status, 462 GC samples were divided into four groups: G1 (38 samples with a survival period of >5 years), G2 (115 samples with a survival period of <5 years and death), G3 (135 samples with a survival period of <5 years but survival), and G4 (174 samples without survival information) (see Supplementary Materials 2.6 for details).

Next, we randomly selected 95% of cases from the GC168 cohort to build a random forest training model and screened a better model with a training AUC of more than 0.9 (Fig. S6A). Based on this, 19 predictive biomarkers were selected (Table S5) with a frequency greater than 50% in the total number of tests (Fig. S6B). Notably, these biomarkers involved 14 genes, namely, *TP53*, *APC*, *PIK3CA*, *NOTCH2*, *CSMD3*, *CDH11*, *ATRX*, *KMT2C*, *MUC16*, *SETD2*, *SRGAP3*, *SETBP1*, *NIN*, and *DCC*, of which at least 50% were involved in the occurrence and development of GC.^5^ Meanwhile, we found that CCA of *MUC16* mutation on SBS44∗ or SBS18∗ also played an important role in the prediction model (Fig. S6B, C). The model was used to predict all cases in the GC168 cohort and obtained a good result (Fig. 1F, AUC: 0.9194, 95% CI: 0.8357–1). We then used the dataset from the ICGC123 cohort to evaluate the model and still reached a good level (Fig. 1G, AUC: 0.857, 95% CI: 0.7148–0.9992). Furthermore, to test the adaptability of the model, we used the mutational signatures obtained by the independent decomposition of the GC168 and ICGC123 datasets to construct a test dataset for analysis. We found that the model obtained approximately 90% accuracy under the GC168 cohort data (Fig. 1H, AUC: 0.894, 95% CI: 0.797–0.991), and the AUC predicted value calculated from the ICGC123 cohort was higher than 0.8 (Fig. 1I, AUC: 0.8153, 95% CI: 0.6326–0.9981).

For a more comprehensive analysis, we constructed independent prediction models for the FYSR of SBS44 and SBS18 using the same method. The AUC of SBS18 was 0.87 and 0.80 for the GC168 and ICGC123 datasets (Fig. S7A), respectively. The AUCs of SBS44 were 0.86 and 0.82 for the GC168 and ICGC123 datasets (Fig. S7B), respectively, and all were lower than those of SBS44&18. Additionally, we performed the same analysis on clinical information prognostic factors, such as MSI status, Lauren types, and clinical stages (Supplementary Material 3.4) that we discovered. The results showed that the predictive effect was not ideal, with an AUC of less than 0.8 (Fig. S8A, B), and the final predictive biomarkers used were Lauren types and clinical stages (Fig. S8C). In addition, when SBS18 and SBS44 were combined with these clinical information prognostic factors to construct the predictive models for assessment, it was found that the effects were not as good as those of SBS44&18 alone (Fig. S9).

In summary, the prediction model of FYSR constructed based on the relationship between genes and prognosis-related mutational signatures can obtain better prognosis evaluation results and has a certain degree of auxiliary value for clinical medical treatment. Additionally, it should be feasible to advance other tumors in accordance with the model scheme in this work if the clinical information for those tumors is complete (see supplementary material for details).

## Author contributions

Zhenzhang Li and Wen Luo: conceptualization, methodology, investigation, software, writing - original draft. Lingqing Xu and Shaoan Zhang: methodology, investigation, resources. Xiaohong Xu, Chunyu Hou, and Xubei Peng: formal analysis, validation. Shiju E and Janak L. Pathak: formal analysis. Shizhen Zhang and Jiawei Liu: validation. Yang Li and Linhai Li: supervision, writing - review & editing, project administration.

## Conflict of interests

The authors declare to have no conflict of interests.

## Funding

This work was financially supported by the Science and Technology Planning Project of Guangzhou, Guangdong, China (No. 006259497026); the Young Creative Talents of Department Education of Guangdong, China (Natural Science, No. 2019KQNCX067); the National Natural Science Foundation of China (No. 52172083); International Science & Technology Cooperation Program of Guangdong, China (No. 2021A0505030078); Guangzhou Key Research and Development Program (China) (No. 2023B03J1239); Program for Innovative Research Team in University of Education System of Guangzhou, China (No. 202235404).

## Data availability

Relevant clinical data and analysis process data of this work can be obtained in Supplementary Table S1 and Supplementary Tables S2–7, respectively.

## Code availability

The *RNMF* framework and the CCA model calculation are available on GitHub at https://github.com/zhenzhang-li/RNMF. The FYSR prediction model calculation and example are available on GitHub at https://github.com/zhenzhang-li/FYSR-GC.

## References

[1] Tate J.G., Bamford S., Jubb H.C. (2019). COSMIC: the catalogue of somatic mutations in cancer. Nucleic Acids Res.

[2] Li Z., Liang H., Zhang S., Luo W. (2022). A practical framework RNMF for exploring the association between mutational signatures and genes using gene cumulative contribution abundance. Cancer Med.

[3] Xing R., Zhou Y., Yu J. (2019). Whole-genome sequencing reveals novel tandem-duplication hotspots and a prognostic mutational signature in gastric cancer. Nat Commun.

[4] Svetnik V., Liaw A., Tong C., Culberson J.C., Sheridan R.P., Feuston B.P. (2003). Random forest: a classification and regression tool for compound classification and QSAR modeling. J Chem Inf Comput Sci.

[5] Wang J., Shao X., Liu Y. (2021). Mutations of key driver genes in gastric cancer metastasis risk: a systematic review and meta-analysis. Expert Rev Mol Diagn.

