# Supplementary material for: AI-driven analysis establishes the single base substitution signatures as personalized prognostic predictors for five-year survival of gastric cancer
Source: Genes Dis. 2023 Jul 13;11(4):101030. doi: 10.1016/j.gendis.2023.05.021 (PMC10943050; doi:10.1016/j.gendis.2023.05.021)
Supplement: Multimedia component 1 [file mmc1.pdf]

Supplementary Material

Introduction, Materials and Methods, Supplementary Results, Discussion and Supplementary Figures.

AI-driven analysis establishes the single base substitutions signatures as personalized prognostic predictors for five-year survival of gastric cancer

Zhenzhang Li[a, b,1], Lingqing Xu[a,1], Wen Luo[b,1], Shaoan Zhang[a,b], Chunyu Hou[d], Xiaohong Xu[c], Xubei Peng[b], Shiju E[f], Janak L. Pathak[e], Shizhen Zhang[c], Jiawei Liu[a], Linhai Li[a,*], Yang Li[a,*]

[a] School of Biomedical Engineering, The Sixth Affiliated Hospital, Guangzhou Medical University, Guangzhou, 511436, China.

[b] College of Mathematics and Systems Science, Guangdong Polytechnic Normal University, Guangzhou, 510665, China.

[c] The Second Affiliated Hospital, Guangzhou Medical University, Guangzhou, 510260, China.

[d] Center for Learning Sciences and Technologies, The Chinese University of Hong Kong, Shatin, Hong Kong, China.

[e] Affiliated Stomatology Hospital of Guangzhou Medical University, Guangzhou Medical University, Guangzhou, 510182, China.

[f] International School of Photonics, Cochin University of Science and Technology, Kochi 682022, Kerala, India.

***Corresponding Author**: **Yang Li**, **Linhai Li**, The Sixth Affiliated Hospital, School of Biomedical Engineering, Guangzhou Medical University, Xinzao, Panyu District, Guangzhou, 511436, China. **Email**: lychris@sina.com (Y. L.); mature303@126.com (L. L.).

[1]These authors contributed equally to this work.

1. Introduction

Five-year survival rate (FYSR) is an important clinical indicator to evaluate the effectiveness of surgical treatment for cancer patients.[1,2] It is also a significant reference for doctors to set prognostic therapeutic schedules including frequency of radiotherapy or chemotherapy, type of medication and period of physical reexamination, *etc* when the patient is discharged. According to the data of American Cancer Society[3], FYSRs of worldwide top four malignant tumors in terms of mortality including lung cancer, liver cancer, gastric cancer (GC), and colorectal cancer are 17%, 26%, 32%, and 64%, respectively. To improve prognosis survival, their standardized therapeutic schedules are usually more complex than those of other tumors.

Accordingly, an accurate and reasonable prediction for FYSR can definitively control the predictive information about tumor prognostic biology and essentially improve the survival of cancer patients. Unfortunately, the reality is still grim. Current FYSR usually uses neoplasm staging as a clinical adjacent point. Since the neoplasm staging is set on the clinical statistics of survival of cancer patients for five years, this classical statistic method by using an average data as a criterion lacks individuality (For example, the influence of tumor heterogeneity) and reduces the survival rate of cancer patients. Therefore, it is necessary to develop an individual FYSR prediction depending on the individual characteristics such as genetic engineering which may become the effective way to reasonably improve the prognosis of cancer.[4-8]

Recently, artificial intelligence (AI)-driven genetic engineering offered many opportunities for individual precision diagnostic and prognostic therapy of tumors and has already made rapid advances in the above fields.[9,10] In these research paradigms, accuracy is an important parameter to pursue and it depends on the feature extraction of the tumor gene mutation and algorithmic model. Taking gastric cancer (GC) as an example, many researches suggest the signature of single base substitutions (SBS)18, as a kind of mutational signature associated with reactive oxygen species[11], its feature of high mutational load owing to *CDH1* can be used as a potential signature for precise prognosis and evaluation of GC.[12] SBS44, a kind of mutation feature associated with DNA mismatch repair (MMR)[13] deficiency, is another signature waiting to be excavated as a prognostic signature for GC, since its occurrence rate is significantly higher than that of the other signatures including SBS6, SBS18, SBS15, SBS21 and SBS26, *etc*.,[11] when generation of tumors activates DNA mismatch repair mechanism to maintain genomic integrity. In addition, SBS44 more effectively triggers the epigenetic alterations affecting known genes in the repair pathway and/or mutations in genes that plays a role in the repair pathway.

Here, we propose to construct an accurate model of individual FYSR prediction using SBS44*, SBS18* or SBS44&18 (combination of SBS44* and SBS18*) as the background mutational signatures of basic variables; meanwhile, we also customized an AI algorithm, called cumulative contribution abundance (CCA), which can independently evaluate the contribution probability of each gene in each cancer sample to each signature and reduce the interference of mutation load between samples. In contrast to other popular algorithms, such as NTriPath[14], *etc*., the CCA model can better depict the link between genes and mutational signatures, thus ensuring the possibility of achieving the convenient, fast and accurate individual FYSR prediction. We finally acquire the individualized GC FYSR (95% CI from 0.84 to 1) based on the data analysis of the CCA model with hundreds of tumors and validated in three independent states. By comparison

with statistical data of neoplasm staging in the clinic, the accuracy of our prediction is over 90%. To the best of our knowledge, this is the first study using the AI algorithm of CCA to forecast the FYSR of GC and achieved great prediction results.

2. Materials and Methods

2.1. Cumulative contribution abundance (CCA)

$$\begin{cases} C(s,g)=\sum_{k=1}^{M}\sum_{n=1}^{N}\theta(sg,mn), \\ \theta(sg,mn)=\rho(s,mn)\cdot\dfrac{\rho_{mg}^{n}}{\sum_{i=1}^{M}\rho_{ig}^{n}}, \\ \rho(s,mn)=\dfrac{P_{ms}S_{sn}}{\sum_{k=1}^{K}P_{mk}S_{kn}}, \\ \rho_{mg}^{n}=\dfrac{N_{mg}^{n}}{\sum_{i=1}^{G}N_{mi}^{n}}. \end{cases} \quad (1)$$

Somatic mutations in cancer arise from diverse mutational processes, such as replication errors and exposure to endogenous or exogenous DNA damaging agents, each of which generates a characteristic pattern of mutations on the mutational signature.[13,15,16] Therefore, it is critical to study the probability of each somatic mutation contributing to the mutation category. Here, we defined a gene's g contribution to a mutational signature s in tumor n as cumulative contribution abundance (CCA), $C(s, g)$ (**Eq. 1**).

Where $\theta(sg, mn)$ is the contribution of the mutation category m of the gene g to the mutational signature s in the tumor sample n. $\rho(s, mn)$ is the influence of the mutation category m during the mutational process in tumor n. P is the signature matrix. S is the sample contribution matrix. The effect of mutation category m assigned to signature k in tumor n is represented by the unit $P_{mk}S_{kn}$. ρ_{mg}^{n} is the impact factor of the gene g to the mutation category m in the tumor sample n. To calculate ρ_{mg}^{n}, for each mutation type for all the genes in the tumor n, a matrix A of the number of mutations is created.

$$A=\begin{bmatrix} N_{11}^{n} & \cdots & N_{1G}^{n} \\ \vdots & \ddots & \vdots \\ N_{M1}^{n} & \cdots & N_{MG}^{n} \end{bmatrix},$$

Where, G is a symbol for the number of genes. M is a symbol for the number of mutational signatures. N represents the number of genes. By employing this method, we hoped to further the development of fresh perspectives on tumor mechanisms.

2.2. Genome data

The data of this study came from four geographically distinct study cohorts with 462 GC samples (**Table S1**). *Oncotator*[17] annotated all somatic mutations, including short insertion/ deletions and single-nucleotide substitutions. These investigations also produced clinical data (**Table S2**). Only the ICGC and GC168 cohorts have access to patient follow-up and vital statistics, nevertheless.

2.3. Identification of hypermutation samples and significantly mutated genes

We used dynamic programming to apply the most optimal k-means clustering to each sample's tumor mutation load. In this way, the distance within each class can be minimized and the distance between classes can be maximized. We ran this approach with various cluster counts ranging from 1 to 9, taking into account that the precise number of clusters is unknown. The silhouette coefficient is used to determine the ideal number of clusters. Finally, a precise cluster number of 2 was chosen. Then, we separated the 462 GC samples into two groups for further analysis in *R* package *Ckmeans.1d.dp*.[18] Through observation and comparison, we consider the group with a large number of mutations as a hyper-mutated group and the rest as a regular-mutated group. Moreover, the significance of non-silent mutations in a gene with a background mutation rate calculated by silent mutations, while accounting for other confounding covariates, may be quantified by an algorithm called *MutSigCV*[19,20], which is how we also discovered significantly mutated genes.

2.4. Feature extraction and CCA calculation model

From the genomic data of GC samples (n = 562), we extracted mutational signatures using the *RNMF* framework.[19] The software can automatically calculate the ideal number of SBS signatures because it was built on an optimized non-negative matrix factorization.The variation matrix V was factored into the two nonnegative matrices P and S, where P stands for mutational processes and S for the relationship in each sample that denotes the quantity of somatic mutations assigned to the extracted relevant signature. Then, the COSMIC - Catalogue Of Somatic Mutations In Cancer was used to do a cosine similarity analysis on all SBS signatures in order to compare and annotate them.[21,22] We define that a signature is deemed inactive in this sample if the sample's contribution to it is less than 6%. However, if the contribution reaches more than 20%, this signature is considered to be particularly active in the sample. Finally, we perform a CCA analysis and obtain the CCA matrix data of the gene under mutational signatures in order to investigate the causal connection between genes and mutational signatures.

2.5. Survival analysis

A Kaplan–Meier survival analysis was used to examine the relationship between prognosis and mutational biomarkers. The *R survival* package (2.40-1) was used to do a Kaplan-Meier survival analysis. A P-value of 0.05 or less was considered statistically significant.

2.6. Establishment of five-year survival prediction model

In this study, we mainly searched for some biomarkers associated with a five-year survival rate by random forest algorithm based on these mutational signatures associated with prognosis and the CCA matrix data of cancer-associated genes under them. Here, to establish a prediction model of FYSR, we only select the sample data of G1 and G2 for analysis. And those samples come from the GC168 cohort or ICGC123 cohort. Then we subdivided the 153 samples (G1+G2) according to different cohorts to obtain four datasets, namely G1 (GC168, 25 samples), G2 (GC168, 70 samples), G1 (ICGC123, 13 samples) and G2 (ICGC123, 45 samples). We first split the data (G1 and G2) into 2 independent data sets, and then applied the *R randomForest* package (4.6-7) for analysis. The specific steps are as follows:

FYSR Prediction Model

(I) **Data set partition**: Training set: ***A***; Test set: ***B***.

(II) **Training Set Reconstruction**: Extract 70%, 80%, 85%, 90%, 95%, 100% from each group in training set ***A*** → ***SETA***.

(III) **Expand the feature pool**: ***SETA*** is expanded by 1, 2, 3, and 5 times → ***SETB***.

(IV) **Attribute feature filtering**: Filtering the attribute features mean value of **SETB** > 0.001.

(V) **Build multigroup model sets**: The random forest algorithm is used to train the training set ***SETB***, and build multigroup model sets.

(VI) **Tests and evaluates**: For the model set in (V), each group of models tests and evaluates the entire training set ***A***, and simultaneously tests the data set ***B***.

(VII) **Iterative testing**: Carry out the test steps from (II) to (VI) independently 100 times. After completion, select the parameter model group with a small fluctuation of AUC, lower quantile ≥ 0.7, and median and mean ≥ 0.8.

(VIII) **The final prediction model is selected for evaluation**: It is required that the AUC of SETB and A > 0.9, the AUC of B > 0.85, and the frequency of all selected biomarkers under the current parameters is > 50% in these 100 trials.

Finally, to verify reliability of the model, we carried out the following two-step test. (a) Analyzed the mutational signatures obtained from the decomposition of data set A, first obtained the CCA matrix data, and then evaluated it based on the prediction model in (VIII). (b) Analyzed the mutational signatures obtained

from the decomposition of data set B, first obtained the CCA matrix data, and then evaluated it based on the prediction model in (VIII).

3. Supplementary Results

3.1. The research workflows

In this work, we utilized *RNMF*,[19] a practical *R* package from our earlier research that may initiate the signature decomposition directly from the mutation datasets, to obtain SBS signatures presenting at GC from 6 GC Whole Genome Sequencing (WGS) projects (including combined). Then, we defined a gene's *g* contribution to a mutational signature *s* in tumor *n* as cumulative contribution abundance (CCA). According to the CCA calculated results, we confirmed that SBS44* and SBS18* as important signatures in GC. Subsequently, it was proved that they could be used as prognostic factors for survival of GC and analyzed the mutant genes associated with them. Further after testing, we selected 19 predictive biomarkers involving 14 genes contributing to SBS44&18 for the FYSR modeling. Finally, the FYSR model was validated in multiple independent cohorts. The research workflows to test, validate and identify is shown in **Fig. A1**.

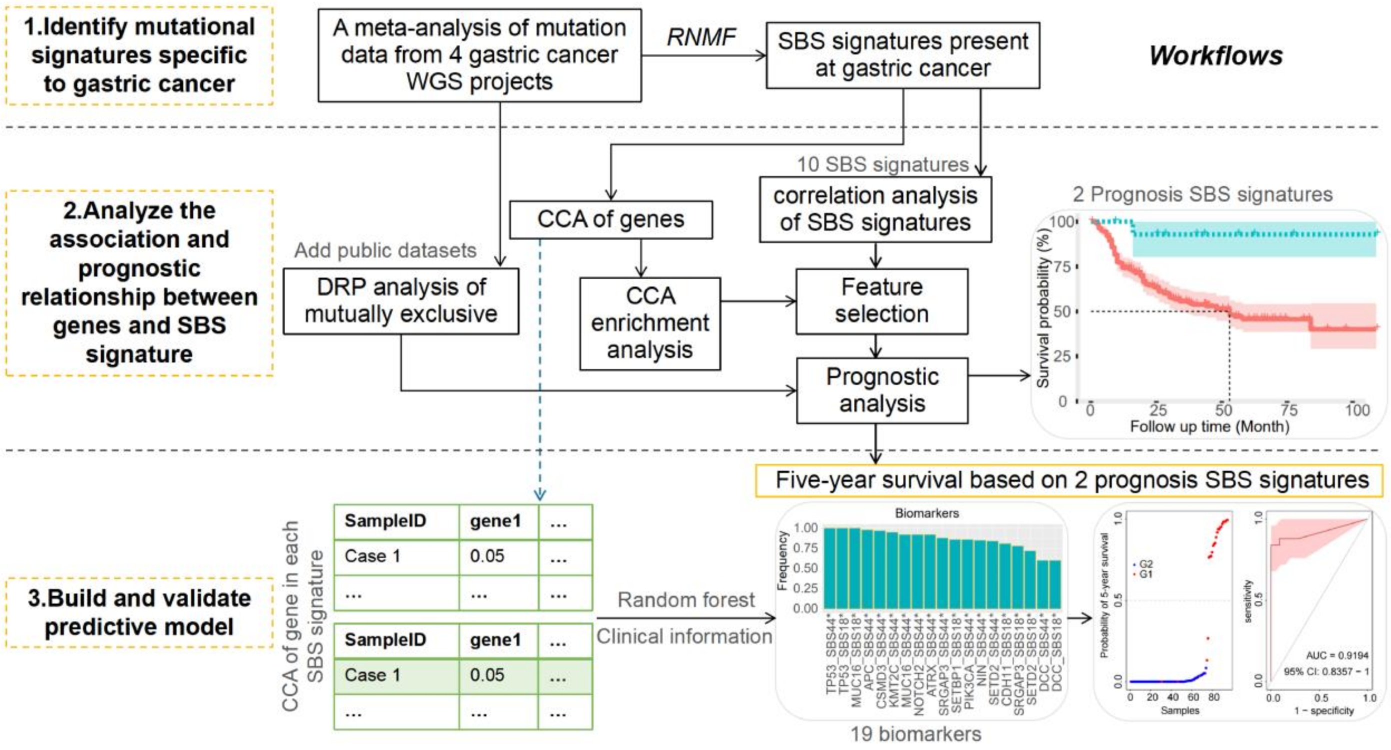

Fig. A1. Workflow of this study. 1: Identify mutational signatures specific to gastric cancer in 462 tumors. 2: Analyze the association and prognostic relationship between genes and signature. 3: Build and validate the predictive model.

3.2. A meta-analysis of gastric cancer sample

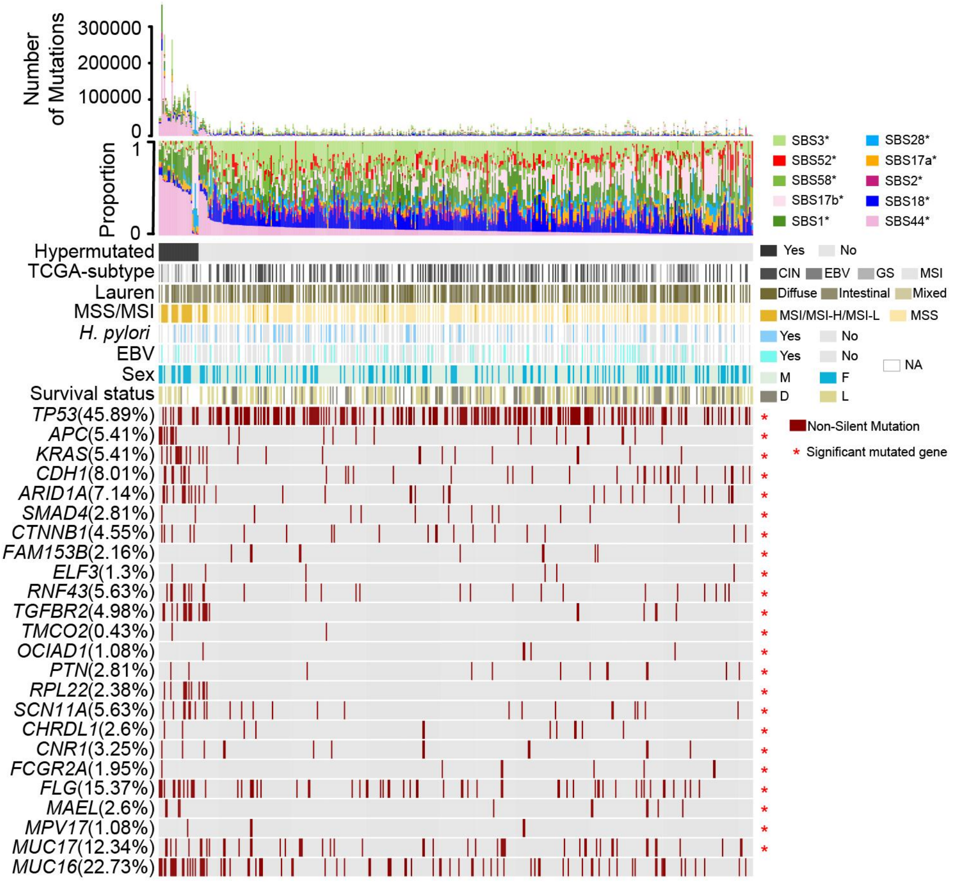

Fig. A2. Overall mutation rates, mutational signature contributions, and mutational status of *MUC16* and other genes of interest in the combined cohort (All cohorts: 462 GCs). Each column represents a tumor. Overall tumor mutation load is shown at the top, followed by the proportion of each of the ten mutational signatures to the overall tumor mutation load (samples are arranged in order of decreasing SBS44* activity), Hypermutated, TCGA-subtype, Lauren, MSI status, H. pylori status, EBV status, Sex and Survival status (D: Dead, L: Live). In the bottom half of the figure, the mutational status of *MUC16* and other genes of interest is shown, with events color-coded by dark red. Significantly mutated genes were marked with red *.

MutSigCV[19,20] was used to identify significantly mutated genes (SMGs), and 22 SMGs were identified in both the whole-genome and -exon region of the regular- mutated group (**Fig. A2, Table S3A**). About 86.4% (19/22) of them were widely reported as driver factors or prognostic factors or involved in the occurrence and development of GC[12,23-25], while the remaining 3 SMGs (*MPV17* (1.08%) , *SCN11A* (5.63%) and *TMCO2* (0.43%)) had not been determined in GC, which required a large number of subsequent studies to verify. Interestingly, during the screening of mutant genes with a non-silent mutation rate greater than 5%, we found that 31.8% of SMGs were significantly enriched in the hyper-mutated group, namely *APC* (5.41%), *KRAS* (5.41%), *RNF43* (5.63%), *CDH1* (8.01%), *ARID1A* (7.14%), *FLG* (15.37%) and *SCN11A* (5.63%) (**Fig. A2, Table S3B**). It is noteworthy that we found transmembrane protein-I gene *MUC16* (22.73%), which is

related to tumor mutation load (TML) and the prognosis of GC patients[26], was significantly enriched in the hypermutated samples.

3.3. Discovery association gene of SBS44* and SBS18* in gastric cancer

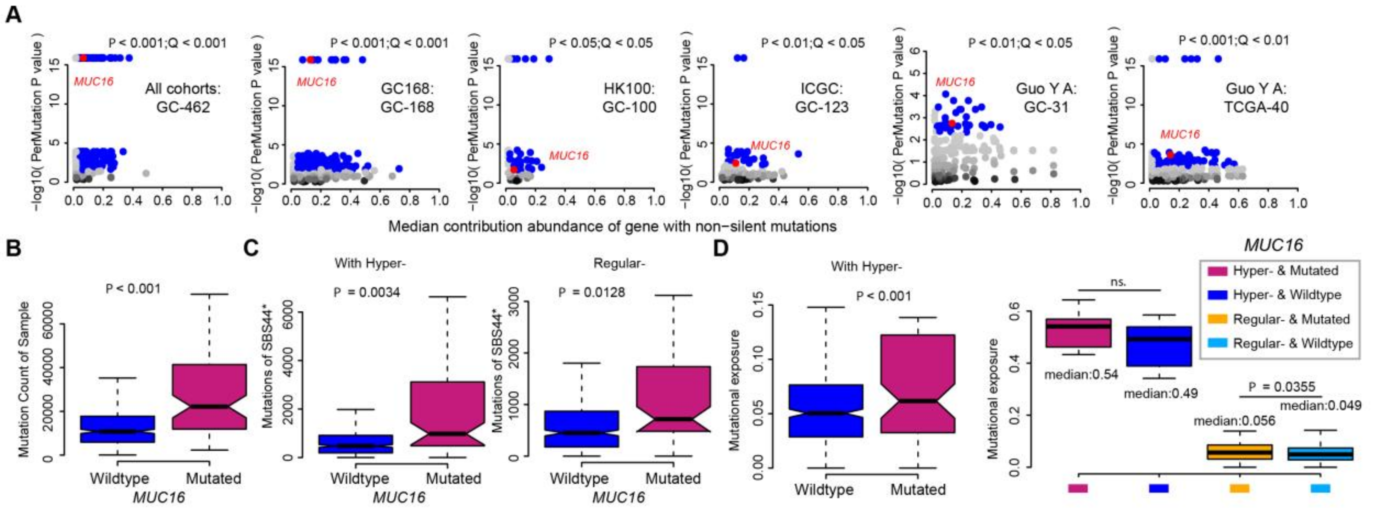

Fig. A3. CCA enrichment analysis identifies an association between somatic *MUC16* mutations and the activity of SBS44* in Gastric Cancer. (A) Here, we use six datasets (with hyper- mutated cases) to find the association between somatic *MUC16* mutations and the activity of SBS44*. First, the median CCA of each gene in the current signature is calculated, and then the contribution importance of each gene is calculated by the PERMUTATION test to study the association between gene and signature. For genes mutated in >5% of samples, the CCA of genes attributed to SBS44* was compared in tumors with wild-type versus mutated copies of the gene. Genes with FDR q < 0.05 and a median of CCA more than 0.01 are highlighted in blue or red (Only for *MUC16*). Each point represents a cancer-associated gene. (B-C) The boxplot diagrams show the difference in mutation counts of samples and mutations contributed to SBS44* between *MUC16*-mutated and *MUC16*-wildtype GC in different states (With Hyper- and only Regular-), while (D) shows the difference of the mutational exposure of SBS44* between *MUC16*-mutated and *MUC16*-wildtype GC in different states (With Hyper-, only Hyper- and only Regular-). The t-test with two-sided is used here, and ns represents ($P \geq 0.05$).

To further investigate the important SBS44* and SBS18* in GC, a signature enrichment analysis was performed using the CCA model to identify mutant genes associated with increased SBS44* and SBS18* activity. For the SBS44*, we analyzed the combined cohorts (all cohorts) and their respective cohorts (GC168, HK100, ICGC, two datasets of Guo Y A) respectively, and validated the *MUC16* was the only significant gene that was present in all cohorts (Benjamini-Hochberg false discovery rate (FDR) q<0.05, p<0.05. **Fig. A3A**). Concurrently, we found that the mutation burden of samples containing non-silent mutations of *MUC16* was significantly increased (median, 22,203 vs 10,817, p<0.001. **Fig. A3B**), which further confirms that mutation of *MUC16* was also related to the tumor mutation burden (TMB) in GC.[26-29] Furthermore, the number of mutations that contributed to SBS44* was significantly increased in tumors which harbored *MUC16* non-silent mutations (With Hyper-, median , 978 vs 475, p=0.0034; Only Regular-, median, 715 vs

450, p=0.0128. **Fig. A3C**). More significantly, we found that tumors carry *MUC16* non-silent mutations gain greater benefits to contribute to SBS44* (With Hyper-, median, 0.062 vs 0.050, p<0.001; Only Regular-, median, 0.056 vs 0.049, p=0.0355. **Fig. A3D**). In conclusion, these findings clearly show that *MUC16* mutations are related with a large increase in SBS44* activity, even though SBS44* activity is likely present in both subtype (*MUC16* wild-type and mutant) GC tumors. Similarly, for the SBS18*, we found that *CDH1* mutation is significantly associated with SBS18* activity (**Table S4A-F**), which was also confirmed by the existing research.[12] Tumors who carried *CHD1* mutations show higher mutational exposure of SBS18*, especially in the Regular- (Median of mutated vs. wildtype: 0.284 vs. 0.141, p<0.001, **Fig. S4B**). But interestingly, mutations of these samples contributed to SBS44* were significantly reduced (**Fig. S4C**), as well as their mutational exposure of SBS44* and mutation burden (**Fig. S4D, E**).

In addition, when analyzing of other signatures based on the CCA model, we noticed an interesting phenomenon that the CCA of *CDH1* is lower in SBS17b* (Only Regular-, **Fig. S4F**). Moreover, we found that there was a significant negative correlation between the mutational exposure of SBS17b* and SBS18* in GC (**Fig. S4G**, P<0.001, R=-0.6). It is noteworthy that tumors that harbored *CDH1* mutation get rather poor results of contributed mutation burden and mutational exposure of SBS17b* (**Fig. S4H**, P<0.001), indicating a negative link between *CDH1* mutations and SBS17b* in GC. According to this, we surmised that SBS17b* may not be a predictor for the survival prognosis of GC.

The occurrence of tumors is mainly the result of a large number of mutations in the genome and the accumulation of such mutations. And those mutations do not occur evenly over the genome.[30] Tumors usually carry one or more driving factors, or significantly mutated genes (SMGs) or high-frequency mutated genes, which play an important role in tumor progression. Whether these genes are independent or cooperative has certain value for tumor research. In previous studies, dimension reduction permutation (DRP)[31] was used to explore the systematic patterns of molecular co-occurrence and exclusivity between the genes mutation, to study the potential association within GC genomic targets, in order to obtain ideas for treatment. Here, we obtained 279 mutually exclusive or symbiotic patterns among SMGs in GC (**Table S6**). Take *MUC16* and *CDH1* as an example, we found that *MUC16* is a frequently mutated gene in GC (**Fig. A2**), and it exhibited mutually exclusive with *CDH1* (**Fig. A4A-D, Table S7**). Therefore, we have a hypothesis that these two genes and their associated signatures may be two different and important patterns in the pathogenesis of GC, which need to be verified by subsequent scientific experiments. And then think deeply, how to calculate the

probability of mutant genes contributed to all signatures remains a crucial issue for better understanding of cancer development.

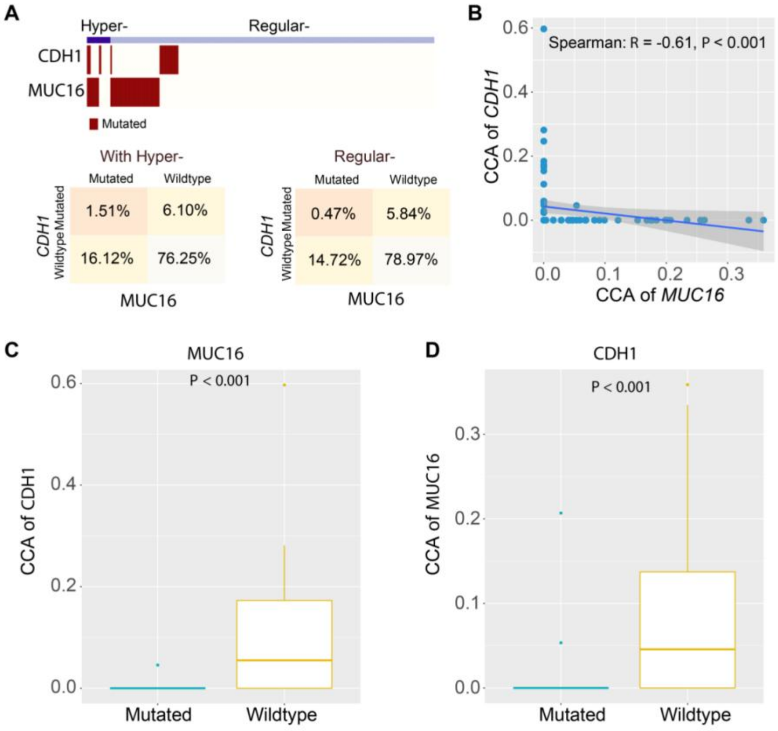

Fig. A4. Molecular exclusivity pattern between *MUC16* and *CDH1*. (A) Statistics of non-silent mutant samples of *CDH1* and *MUC16*, top, mutated samples marked by dark-red, hyper-mutated cases marked by dark-blue. Bottom, showing two cases (With Hyper- and Regular-) of the mutated proportions of *CDH1* and *MUC16* (mutated vs. wildtype). (B) The correlation model of CCA analysis under SBS44* adopts Spearman coefficient. Each light blue dot represents a regular cohort of samples with mutations in the gene *CDH1* or *MUC16*. (C, D) Boxplots showing the difference of the CCA of *CDH1* (*MUC16*) under SBS44* in the mutant group of *MUC16* (*CDH1*) and the wildtype group. The samples analyzed were those from the Regular-cohort and contained mutations in the genes *CDH1* or *MUC16*. The t-test with two-sided is used here.

3.4. Clinical information prognostic factors for survival in gastric cancer

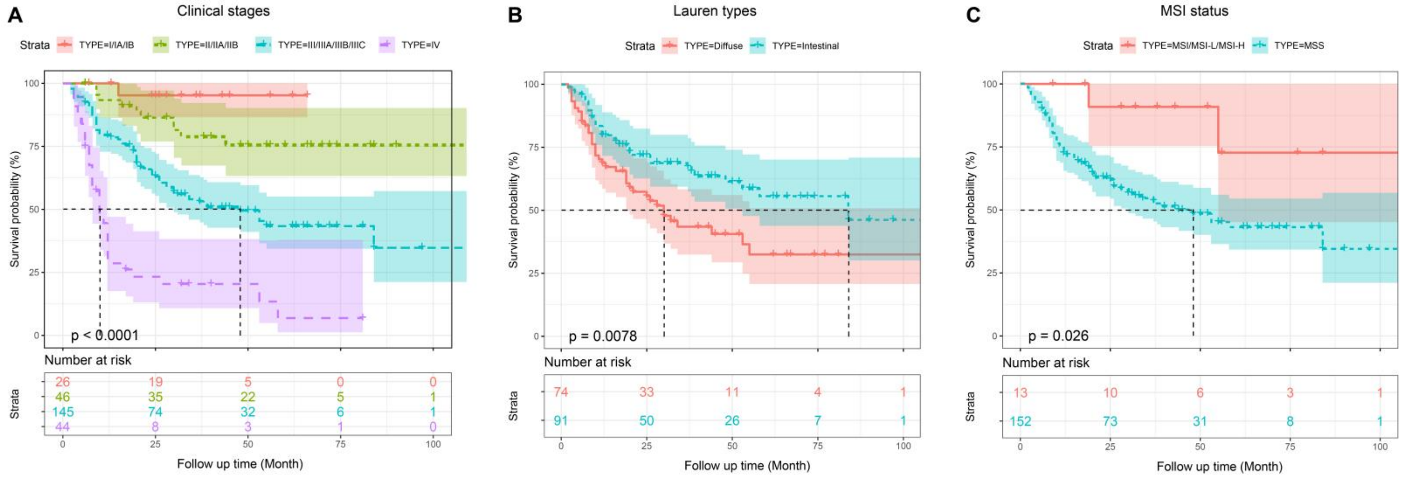

Fig. A5. Prognosis of clinical information in Gastric Cancer. (A)-(C) Kaplan-Meier survival analysis stratified by mutational exposure of clinical stages Lauren types and MSI status, respectively.

In addition to mutational signatures, we also analyzed clinical information in the analysis of prognostic factors for GC. It was found that in addition to SBS18 and SBS44, there were three other prognostic factors, such as Lauren types (**Fig. A5A**), Clinical stages (**Fig. A5B**) and MSI status (**Fig. A5C**). However, these three prognostic factors all belong to clinical information and are not numerical variables.

3.5. Relationship Analysis of SBS44 and SBS18

During the analysis of SBS44 and SBS18, we naturally consider whether it is possible to further integrate SBS44 and SBS18 into one signature? Here, we don't think it's a good choice to integrate SBS44 and SBS18 further into one signature for the following two reasons: (a) From the perspective of NMF algorithm decomposition, the selection of the number of signatures is based on the measure that the stability of the model decomposition reaches the optimal level and the classification error reaches the minimum. In addition, previous studies[11,12, 32] have reported that these two signatures are also independent of each other in GC. In this work, we use RNMF[19] software based on NMF algorithm to independently decompose different data sets (**Fig. A2**), and still decompose these two signatures under the optimal number of signatures, which indicates that these two signatures should be different. (b) Typically, during the course of cancer development, mutational processes from different aetiologies are active. They can be recognized by using mutational signatures because of their distinctive mutational pattern and particular action on the genome. Consequently, mutational signatures are a collection of active mutational processes.[11,21] They play an important role in the biological significance of tumor research. Such as SBS44, which is one of seven mutational signatures associated with defective DNA mismatch repair and microsatellite instability (MSI), was found to be associated with *MUC16* mutations. For SBS18, which is associated with defective base excision repair due to *MUTYH* mutations, was considered that it is related to *CDH1* mutations.[12] Interestingly, we found that *MUC16* exhibited mutually exclusive with *CDH1* (**Fig. A4**). Moreover, we found that there is no positive correlation between the sample contribution of SBS44 and SBS18 (**Fig. S7C**). Interestingly enough, we found that these samples with higher SBS44 contribution ($\geq$ 20%) were significantly associated with a better survival outcome in this cohort (**Fig. 1D**). On the contrary, those cases with higher SBS18 contribution ($\geq$ 20%) showed significantly poor prognosis (**Fig. 1E**), which has been reported.[12] From the perspective of biological analysis, it is highly possible that SBS44 and SBS18 have different mutational processes, resulting in their different roles in the occurrence and development of GC. Therefore, we believe that decomposing into two different signatures is more biologically meaningful and valuable than merging them into one signature.

4. Discussion

In this study, using the CCA model to perform a genome meta-analysis on 462 GC WGS data from 4 previous publications, we discovered SBS44/18 as two significant mutational signatures in GC. Through CCA model analysis, we confirmed that SBS18* is a prognostic factor for survival of GC,[11,12] and it displays a strong link to *CDH1* mutation. Simultaneously, another prognostic factor, SBS44*, was showed to be associated with *MUC16* mutations. Then we built a machine learning model using these associations as the background characteristics of basic variables to predict the FYSR of GC, with an AUC of 0.9194.

The occurrence of tumors is mainly the result of a large number of mutations in the genome and the accumulation of such mutations. And those mutations do not occur evenly over the genome.[30] Tumors usually carry one or more driving factors, or significantly mutated genes (SMGs) or high-frequency mutated genes, which play an important role in tumor progression. Whether these genes are independent or cooperative has certain value for tumor research. In previous studies, dimension reduction permutation (DRP)[31] was used to explore the systematic patterns of molecular co-occurrence and exclusivity between the genes mutation, to study the potential association within GC genomic targets, in order to obtain ideas for treatment. Here, we obtained 279 mutually exclusive or symbiotic patterns among SMGs in GC (**Table S6**). Take *MUC16* and *CDH1* as an example, we found that *MUC16* is a frequently mutated gene in GC (**Fig. 2**), and its exhibited mutually exclusive with *CDH1* (**Fig. S6A-S6D, Table S7**). Therefore, we have a hypothesis that these two genes and their associated signatures may be two different and important patterns in the pathogenesis of GC, which need to be verified by subsequent scientific experiments. And then think deeply, how to calculate the probability of mutant genes contributed to all signatures remains a crucial issue for better understanding of cancer development.

By individually assessing the contribution likelihood of each gene to each mutational signature in each tumor, the CCA model can significantly reduce the interference of mutational burden among samples. This allows it to calculate the CCA of every gene to every mutational signature. In turn, to better understand the linkage of cancer development, the correlation between the genes and mutational signatures can also be obtained. In-depth combination research on CCA of mutant genes contributed to mutational signatures may provide prognosis and prediction information about tumor biology, which can guide the clinical nursing and improve the treatment options for cancer patients.[4-7,35] Additionally, it is believed that mutational signatures result from many alterations in pathway component events[21,36], and that their evaluation as a classifier may be

more insightful than particular clinical or molecular signatures. Therefore, mining the linked genes' internal relationships and mutational signatures will open the door to cancer prognostic analysis.

At present, the FYSR prediction is usually based on tumor stage, but this classical statistical method lacks consideration of the influence of tumor genomes, such as the tumor heterogeneity. Understanding the individual genome signature and formulating personalized treatment strategies will helpful to improve the quality of life for patients after treatment and improve their FYSR. Consequently, we employ the CCA model and characteristic gene variable of single base substitutions (SBS) 44 and 18 signature as scheme frame to construct a model for such FYSR prediction. 19 predictive biomarkers involving 14 genes were selected to build the FYSR prediction model using a random forest model. These genes contained *TP53*, *APC*, *PIK3CA*, *NOTCH2*, *CSMD3*, *CDH11*, *ATRX*, *KMT2C*, *MUC16*, *SETD2*, *SRGAP3*, *SETBP1*, *NIN*, and *DCC*, of which at least 50% were involved in the occurrence and development of GC.[20,31,32] Therefore, our work offers a therapeutically helpful tool for enhancing the management of GC prognosis.

In addition, the prediction model in this work only analyzes GC as a case, mainly limited by the available sample data information, because most solid tumor data lacks effective clinical information, such as prognosis information. Due to the collected data of GC, which has the follow up information and relatively complete mutations that detected from whole-genome sequencing data, the prediction scheme in this work has been effectively verified. If the clinical information of the corresponding sample data on the other tumors is complete, our prediction model scheme should be able to be extended to that tumor. Of course, although most of the mutational signatures are shared by multiple cancers, different cancers have their preferred mutation processes[11-12], so SBS44 and SBS18 signatures are not necessarily survival prognostic factors for other tumors. However, it is feasible to look for pertinent prognostic factors and build prediction models in accordance with the model framework presented in this work. As a result, the prediction model construction technology proposed in our article should be continuously expandable.

References

[1] Spigel DR, Faivre-Finn C, Gray JE, et al. Five-Year Survival Outcomes From the PACIFIC Trial: Durvalumab After Chemoradiotherapy in Stage III Non-Small-Cell Lung Cancer. J Clin Oncol. 2022;40(12):1301-1311.

[2] Zhang X, El-Serag HB, Thrift AP. Predictors of five-year survival among patients with hepatocellular carcinoma in the United States: an analysis of SEER-Medicare. Cancer Causes Control. 2021;32(4):317-325.
[3] Cancer Types | Cancer Resources | American Cancer Society. Accessed 2019. https://www.cancer.org/cancer/all-cancer-types.html.
[4] Sparano JA, Gray RJ, Makower DF, et al. Adjuvant Chemotherapy Guided by a 21-Gene Expression Assay in Breast Cancer. N Engl J Med. 2018;379(2):111-121.
[5] Karapetis CS, Khambata-Ford S, Jonker DJ, et al. K-ras mutations and benefit from cetuximab in advanced colorectal cancer. N Engl J Med. 2008;359(17):1757-1765.
[6] Hoshida Y, Villanueva A, Kobayashi M, et al. Gene expression in fixed tissues and outcome in hepatocellular carcinoma. N Engl J Med. 2008;359(19):1995-2004.
[7] Feller-Kopman D, Liu S, Geisler BP, DeCamp MM, Pietzsch JB. Cost-Effectiveness of a Bronchial Genomic Classifier for the Diagnostic Evaluation of Lung Cancer. J Thorac Oncol. 2017;12(8):1223-1232.
[8] LeVasseur N, Sun J, Fenton D, et al. Impact of the 21-Gene Recurrence Score Assay on the Treatment of Estrogen Receptor-Positive, HER2-Negative, Breast Cancer Patients With 1-3 Positive Nodes: A Prospective Clinical Utility Study. Clin Breast Cancer. 2022;22(1):e74-e79.
[9] Meggendorfer M, Walter W, Haferlach C, Kern W, Haferlach T. Challenging Blast Counts By Machine Learning Techniques and Genome Sequencing for Discriminating AML and MDS. Blood. 2019;134:4663.
[10] Troyanskaya O, Trajanoski Z, Carpenter A, Thrun S, Razavian N, Oliver N. Artificial intelligence and cancer. Nature Cancer. 2020;1(2):149-152.
[11] Alexandrov LB, Kim J, Haradhvala NJ, et al. The repertoire of mutational signatures in human cancer. Nature. 2020;578(7793):94-101.
[12] Xing R, Zhou Y, Yu J, et al. Whole-genome sequencing reveals novel tandem-duplication hotspots and a prognostic mutational signature in gastric cancer. Nat Commun. 2019;10(1):2037.
[13] Peltomäki P. Role of DNA mismatch repair defects in the pathogenesis of human cancer. J Clin Oncol. 2003;21(6):1174-1179.
[14] Cheong JH, Wang SC, Park S, et al. Development and validation of a prognostic and predictive 32-gene signature for gastric cancer. Nat Commun. 2022;13(1):774.

[15] Cancer Genome Atlas Research Network. Integrated genomic analyses of ovarian carcinoma. Nature. 2011;474(7353):609-615.

[16] Dobbs FM, van Eijk P, Fellows MD, Loiacono L, Nitsch R, Reed SH. Precision digital mapping of endogenous and induced genomic DNA breaks by INDUCE-seq. Nat Commun. 2022;13(1):3989.

[17] Ramos AH, Lichtenstein L, Gupta M, et al. Oncotator: cancer variant annotation tool. Hum Mutat. 2015;36(4):E2423-E2429.

[18] Wang H, Song M. Ckmeans.1d.dp: Optimal k-means Clustering in One Dimension by Dynamic Programming. R J. 2011;3(2):29-33.

[19] Li Z, Liang H, Zhang S, Luo W. A practical framework RNMF for exploring the association between mutational signatures and genes using gene cumulative contribution abundance. Cancer Med. 2022;11(21):4053-4069.

[20] Su X, Li Q, Liu S, Cao Y, Xu Y, Yibin W. Clinical Values and Functional Analysis Of KMT2A/B/C/D/E/F in Gastric Cancer by Integrated Bioinformatics Analysis. Research Square, 2021; 1-19.

[21] Alexandrov LB, Nik-Zainal S, Wedge DC, et al. Signatures of mutational processes in human cancer. Nature. 2013;500(7463):415-421.

[22] Kandoth C, McLellan MD, Vandin F, et al. Mutational landscape and significance across 12 major cancer types. Nature. 2013;502(7471):333-339.

[23] He Y, Wang X. Identification of molecular features correlating with tumor immunity in gastric cancer by multi-omics data analysis. Ann Transl Med. 2020;8(17):1050.

[24] Choi JH, Kim YB, Ahn JM, et al. Identification of genomic aberrations associated with lymph node metastasis in diffuse-type gastric cancer. Exp Mol Med. 2018;50(4):1-11.

[25] Guo YA, Chang MM, Huang W, et al. Mutation hotspots at CTCF binding sites coupled to chromosomal instability in gastrointestinal cancers. Nat Commun. 2018;9(1):1520. Published 2018 Apr 18.

[26] Li X, Pasche B, Zhang W, Chen K. Association of MUC16 Mutation With Tumor Mutation Load and Outcomes in Patients With Gastric Cancer. JAMA Oncol. 2018;4(12):1691-1698.

[27] Tate JG, Bamford S, Jubb HC, et al. COSMIC: the Catalogue Of Somatic Mutations In Cancer. Nucleic Acids Res. 2019;47(D1):D941-D947.

[28] Drost J, van Boxtel R, Blokzijl F, et al. Use of CRISPR-modified human stem cell organoids to study the origin of mutational signatures in cancer. Science. 2017;358(6360):234-238.

[29] Yang Y, Zhang J, Chen Y, Xu R, Zhao Q, Guo W. MUC4, MUC16, and TTN genes mutation correlated with prognosis, and predicted tumor mutation burden and immunotherapy efficacy in gastric cancer and pan-cancer. Clin Transl Med. 2020;10(4):e155.

[30] Letouzé E, Shinde J, Renault V, et al. Mutational signatures reveal the dynamic interplay of risk factors and cellular processes during liver tumorigenesis. Nat Commun. 2017;8(1):1315.

[31] Deng N, Goh LK, Wang H, et al. A comprehensive survey of genomic alterations in gastric cancer reveals systematic patterns of molecular exclusivity and co-occurrence among distinct therapeutic targets. Gut. 2012;61(5):673-684.

[32] https://cancer.sanger.ac.uk/signatures/sbs/sbs18/.

[33] Le GM, O'Malley CD, Glaser SL, et al. Breast implants following mastectomy in women with early-stage breast cancer: prevalence and impact on survival. Breast Cancer Res. 2005;7(2):R184-R193.

[34] Helleday T, Eshtad S, Nik-Zainal S. Mechanisms underlying mutational signatures in human cancers. Nat Rev Genet. 2014;15(9):585-598.

[35] Wang J, Shao X, Liu Y, et al. Mutations of key driver genes in gastric cancer metastasis risk: a systematic review and meta-analysis. Expert Rev Mol Diagn. 2021;21(9):963-972.

[36] Nemtsova MV, Kalinkin AI, Kuznetsova EB, et al. Clinical relevance of somatic mutations in main driver genes detected in gastric cancer patients by next-generation DNA sequencing. Sci Rep. 2020;10(1):504.

Supplementary Figures

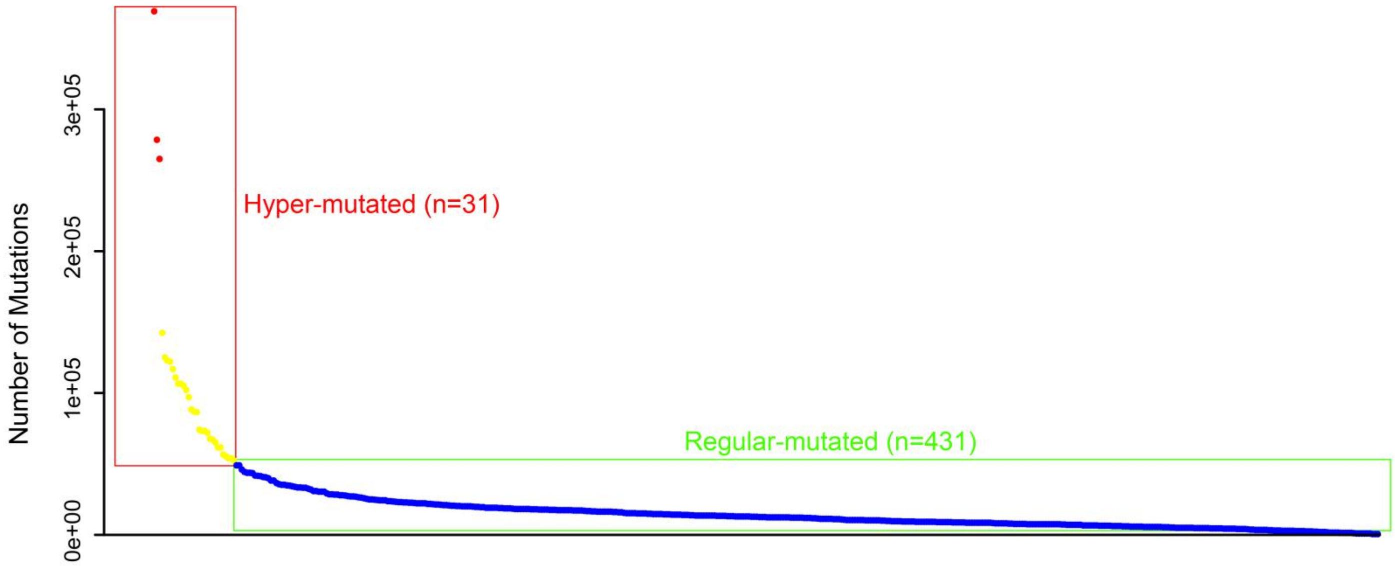

Fig. S1. Tumor mutation load stratified GC samples into the hyper- (red and yellow) and regular- (blue) mutated groups. Each dot represents a case.

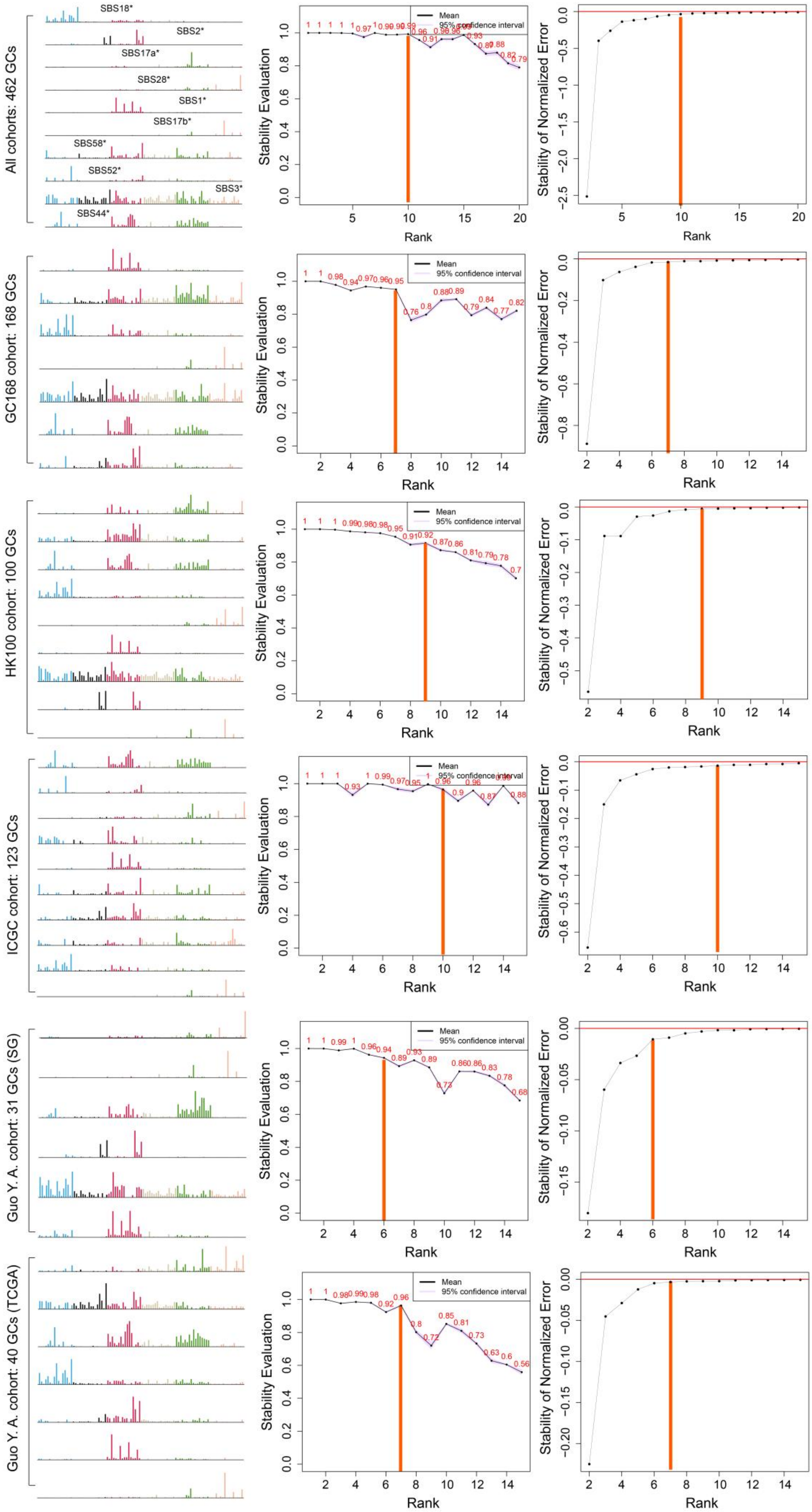

Fig. S2. Deciphering mutational signatures from six dataset. The left side of the picture shows the classifications of 96 mutation types. Each color is used to illustrate the positions of each mutation subtype on each plot. The right side of the picture shows the number of SBS signatures was chose based on strong stability, low error rate and more stable gradient of error. We choose the classification with stability not less than 0.9 as the best number of SBS signatures for final decomposition, in which the vermilion vertical line on each graph indicates the current classification position to be selected. The light purple band represents the confidence region of stability change under the current classification number.

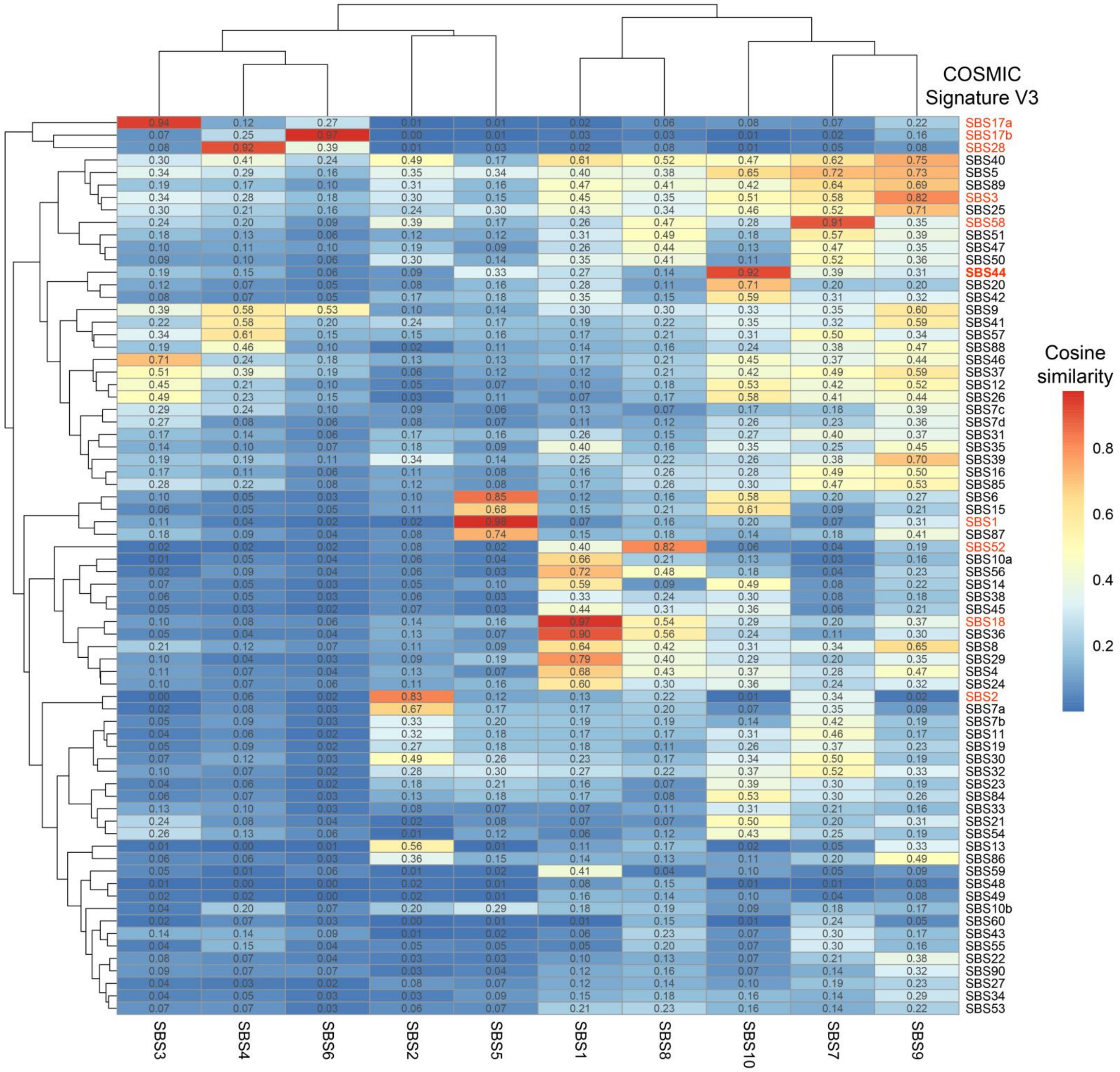

Fig. S3. Heatmap of the cosine similarity between mutational signatures and COSMIC Mutational Signatures (v3.1 - June 2020). The shade of color corresponds to different cosine similarity scores. The SBS signatures with the most similar one is highlighted in red.

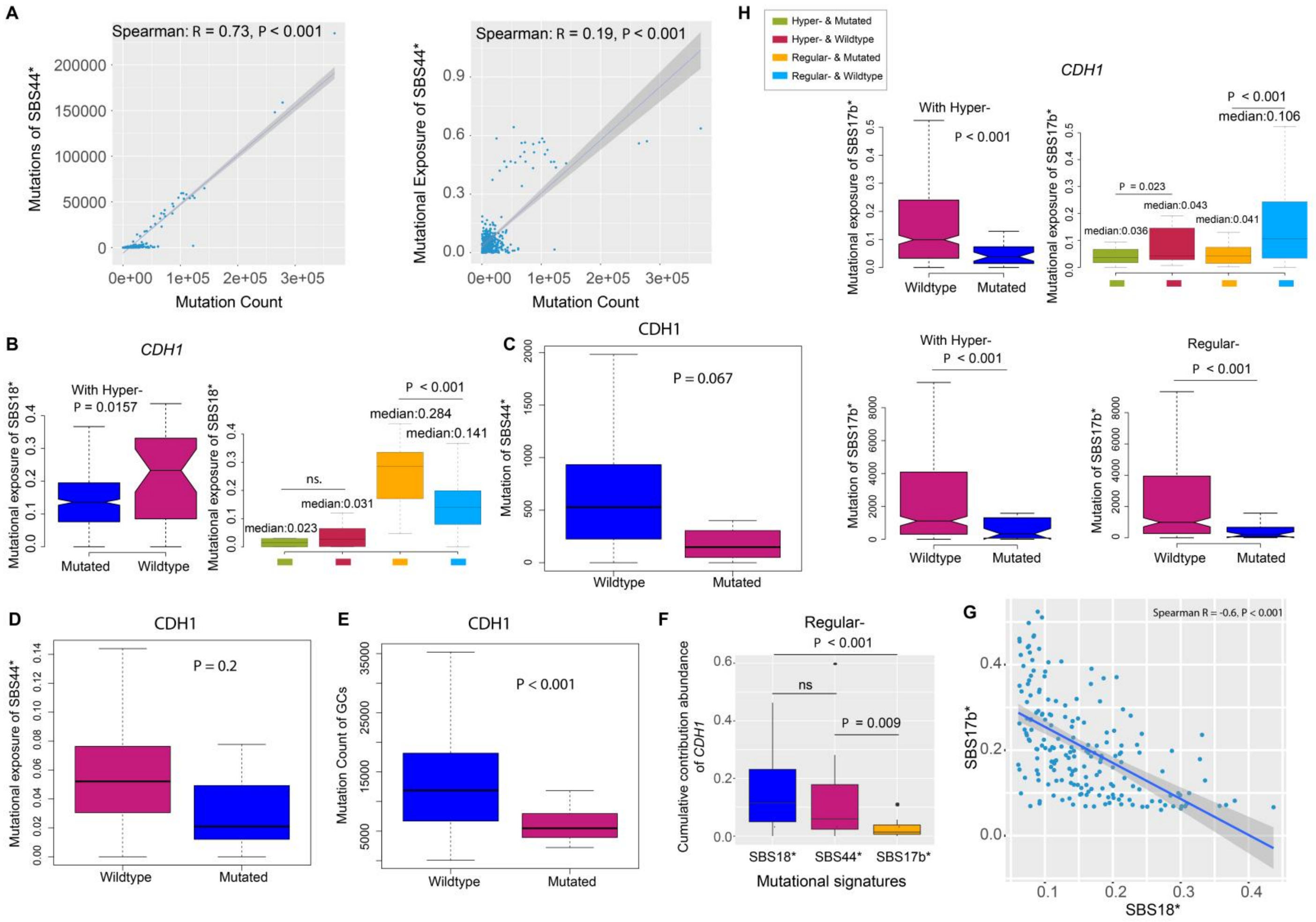

Fig. S4. SBS44 and SBS18 Association Diagram. (A) Spearman correlation coefficient is used to analyze the correlation between the sample's tumor mutation load and the sample's contribution mutation number or contribution degree to SBS44*, where each blue dot represents a sample. **(B-C)** Boxplots show the distribution of the number and degree of contribution to SBS44* for *CDH1* mutated and wildtype samples, respectively. The t-test with two-sided is used here. **(D)** Tumor mutation load compared in different groups of samples (*CDH1* mutated vs. *CDH1* wildtype). The t-test with two-sided is used here. **(E)** Mutational exposure of SBS18* was compared in different groups (With Hyper-, only Hyper- and only Regular-), different colors represent different groups, the statistical test uses t-test with two-sided, the ns represents Not significant, that is, the p-value is greater than or equal to 0.05. **(F)** The CCA of *CDH1* in the Regular- sample is compared in SBS18*, SBS44* and SBS17b*. The statistical test adopts t-test with two-sided, and its ns represents not significant, that is, the p value is greater than or equal to 0.05. **(G)** based on the contribution of the samples to SBS, the correlation between SBS18* and SBS17b* is displayed using the Spearman correlation coefficient, where each blue dot represents a sample. **(H)** The correlation between *CDH1* gene mutation and SBS17b* in gastric cancer. Different colors represent different groups. The statistical test adopts t-test with two-sided, and the ns represents not significant, that is, the p value is greater than or equal to 0.05.

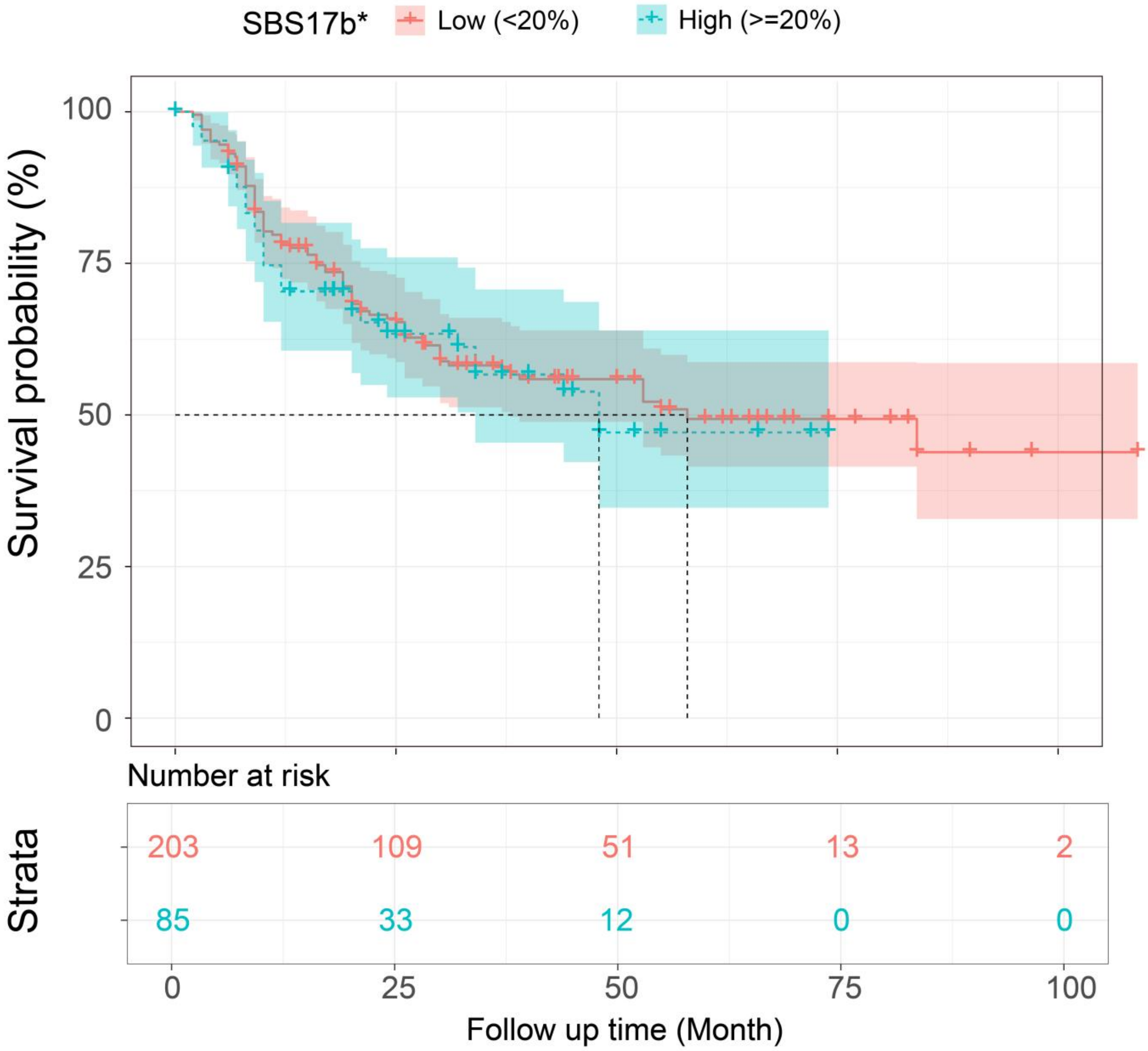

Fig. S5. Kaplan-Meier survival analysis stratified by mutational exposure of SBS17b* status.

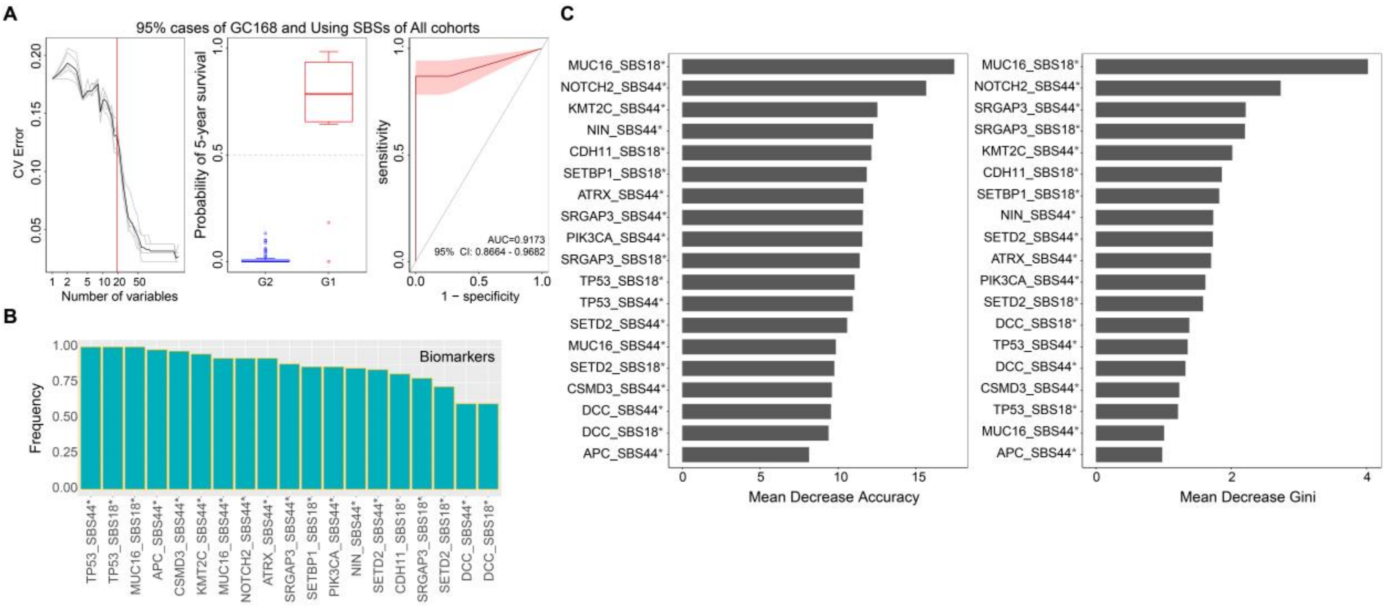

Fig. S6. SBS44 and SBS18 associated gene in survival prognosis of gastric cancer. (A) Distribution of 5 trials of 10-fold cross-validation error in random forest classification of carcinoma as the number of variables increases. The model was trained using CCA of cancer-related genes in 95% cases of the GC168 cohort, and SBSs for model analysis extracting from the combined cohort (All cohorts). The black curve indicates average of the five trials (grey lines). The pink line marks the number of variables. Box-and-whisker plot for the probability of 5-year survival in the cross-validation training set according to the model. Receiver operating curve (ROC) for the training set. The area under the receiver operating curve (AUC) is 91.73% and 95% confidence interval (CI) is 86.64–96.82%. **(B)** Barplot plot showing the frequency of 19 biomarkers associated with GC five-year survival prediction across 100 trials. Each column represents a biomarker. **(C)** Mean Decrease Accuracy and Mean Decrease Gini display of these 19 biomarkers in random forest.

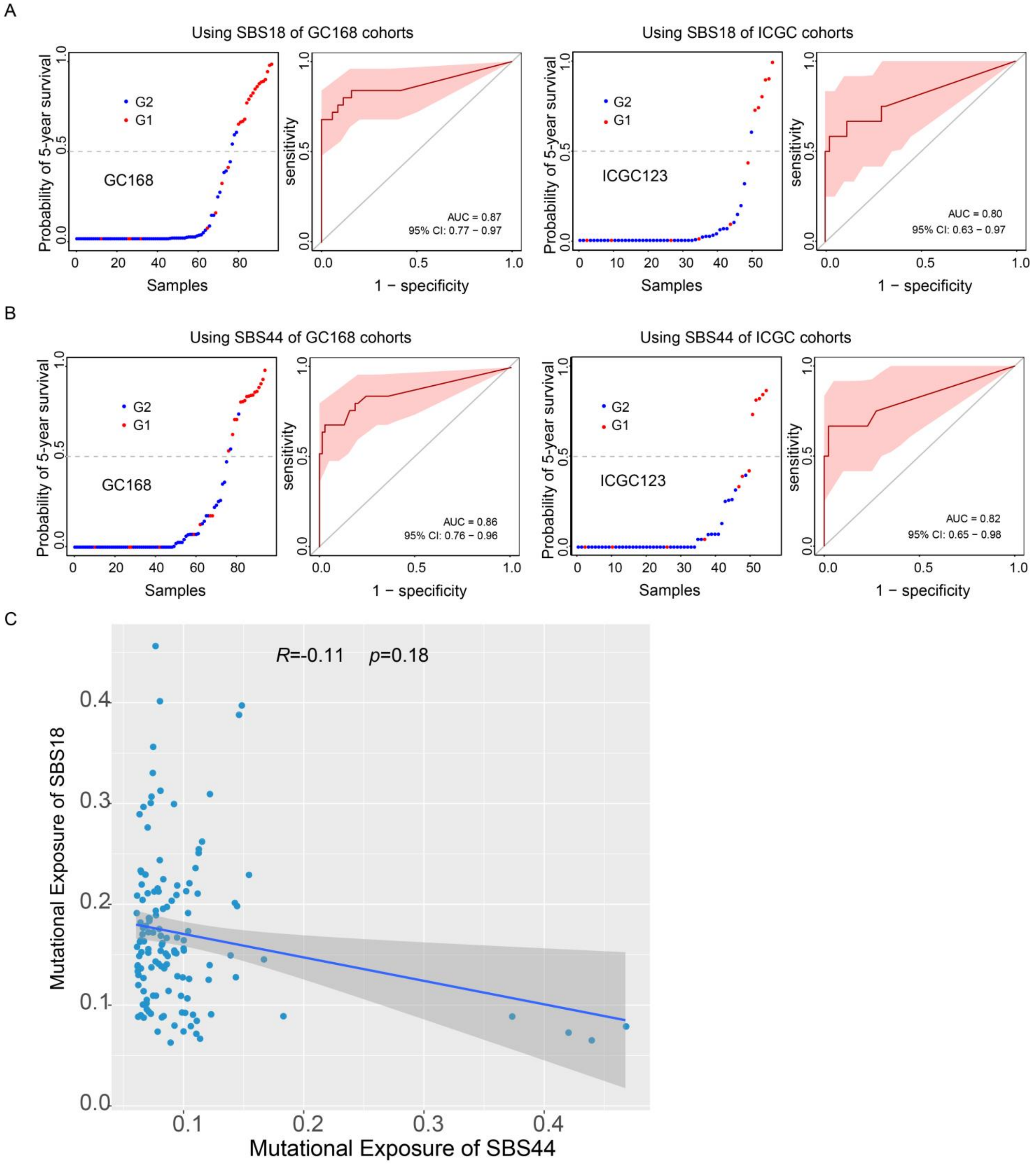

Fig. S7. SBS18 and SBS44 independent classify five years survival in Gastric Cancer. (A) SBS18 classification consisted of G2 (blue) and G1 (red) from the GC168 and ICGC123 cohort, respectively. The area under the receiver operating curve (AUC) is 87% and 95% confidence interval (CI) is 0.77-0.97 (GC168); The area under the receiver operating curve (AUC) is 80.0% and 95% confidence interval (CI) is 0.63-0.97 (ICGC123). **(B)** SBS44 classification consisted of G2 (blue) and G1 (red) from the GC168 and ICGC123 cohort, respectively. The area under the receiver operating curve (AUC) is 86.0% and 95% confidence interval (CI) is 0.76-0.96 (GC168); The area under the receiver operating curve (AUC) is 82.0% and 95% confidence interval (CI) is 0.65-0.98 (ICGC123). G2 refers to the samples with survival of less than 5 years but died, and G1 refers to the samples with a survival of more than 5 years. (**C**) Correlation between SBS18 and SBS44.

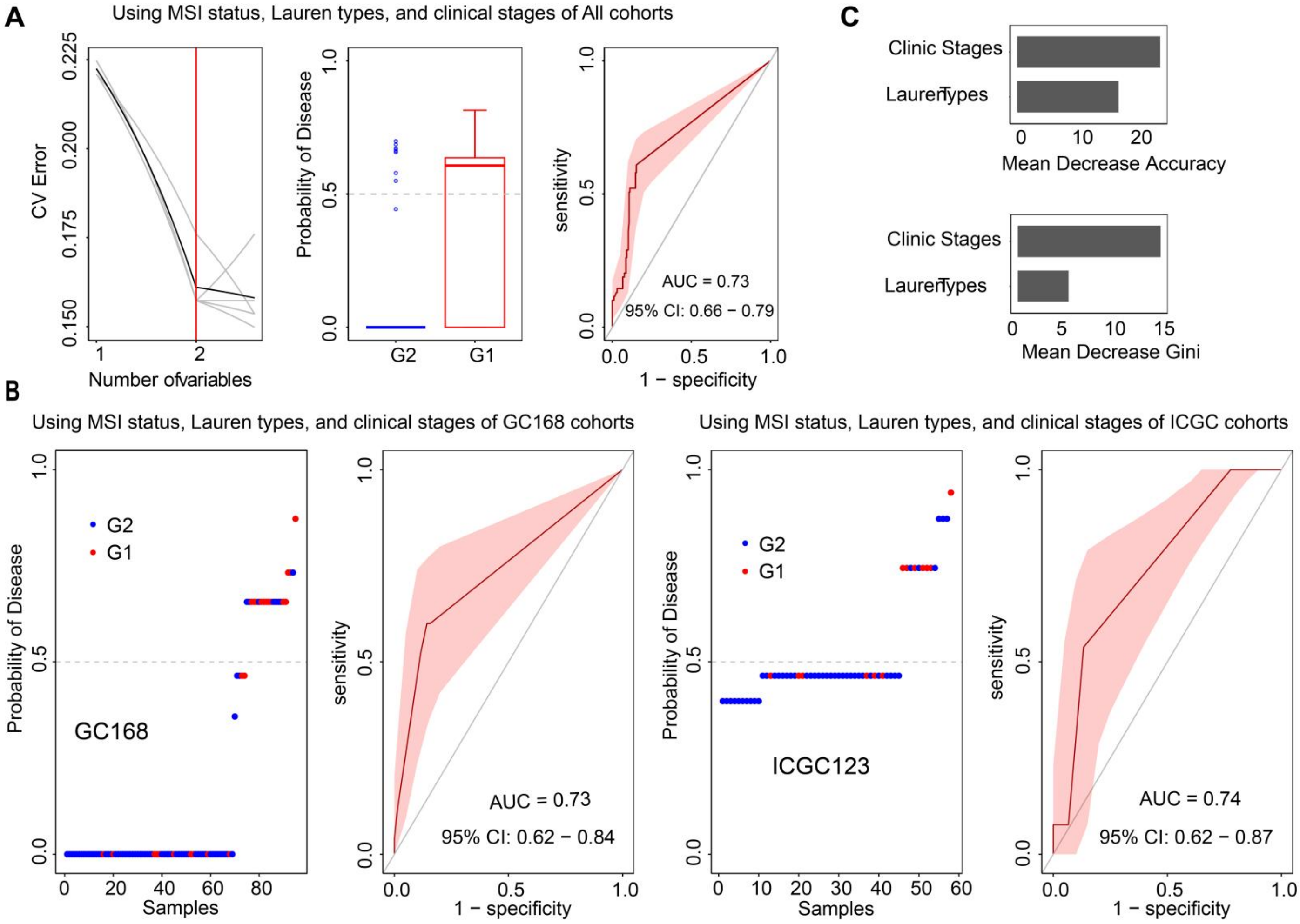

Fig. S8. MSI status, Lauren types and clinical stages associated gene in survival prognosis of gastric cancer. (**A**)Distribution of 5 trials of 10-fold cross-validation error in random forest classification of carcinoma as the number of variables increases. The model was trained using CCA of cancer-related genes in 95% cases of the GC168 cohort, and MSI status, Lauren types and clinical stages for model analysis extracting from the combined cohort (All cohorts). The black curve indicates average of the five trials (grey lines). The pink line marks the number of variables. Box-and-whisker plot for the probability of 5-year survival in the cross-validation training set according to the model. Receiver operating curve (ROC) for the training set. The area under the receiver operating curve (AUC) is 73% and 95% confidence interval (CI) is 0.66–0.79. (**B**) Using MSI status, Lauren types and clinical stages of GC168 cohorts, The area under the receiver operating curve (AUC) is 73% and 95% confidence interval (CI) is 0.62–0.84; Using MSI status, Lauren types and clinical stages of ICGC123 cohorts, The area under the receiver operating curve (AUC) is 74% and 95% confidence interval (CI) is 0.62–0.87; (**C**)

G2 refers to the samples with survival of less than 5 years but died, and G1 refers to the samples with a survival of more than 5 years.

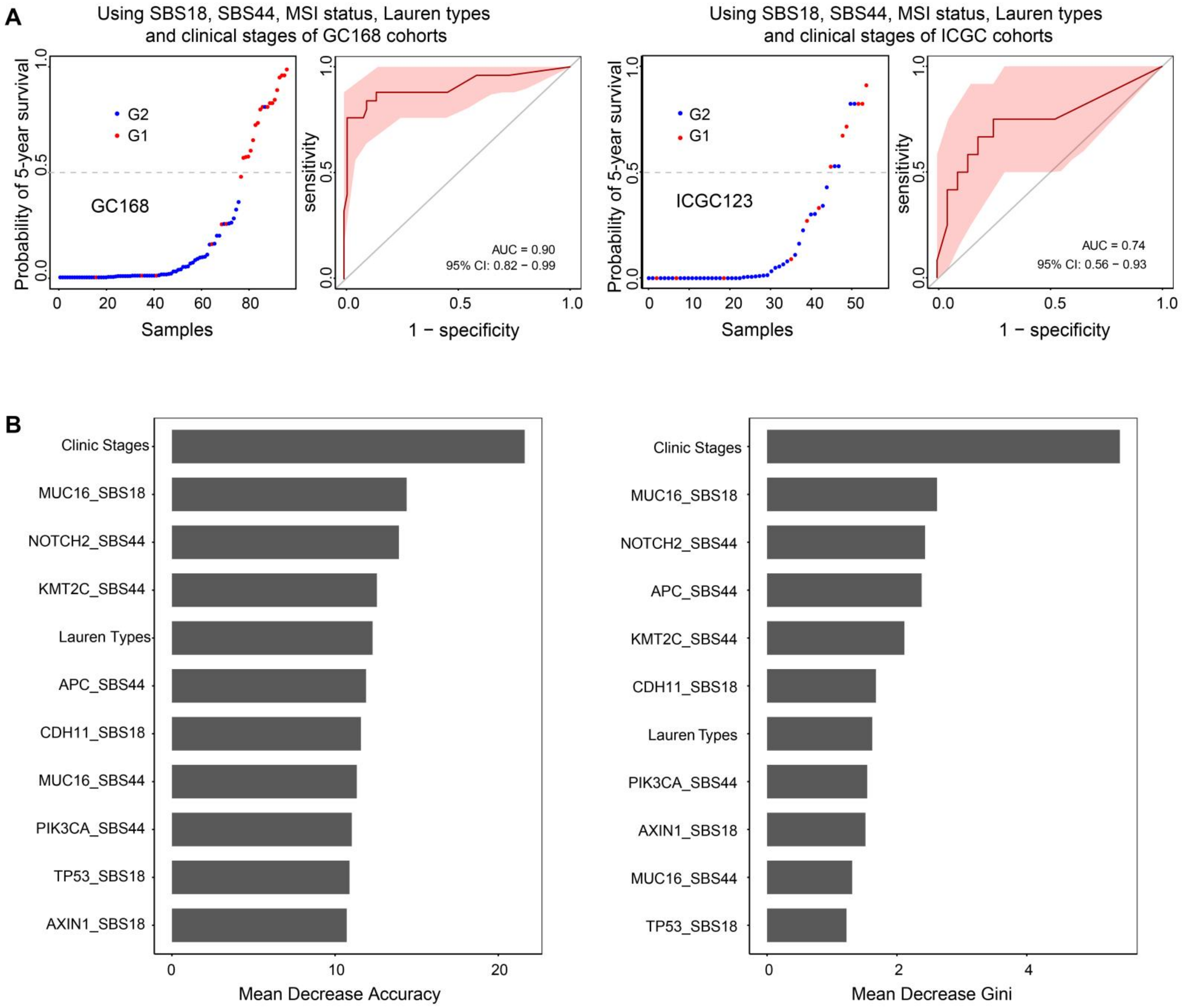

Fig. S9. SBS18, SBS44, MSI status, Lauren types and clinical stages combined classify five years survival in Gastric Cancer. (A) Classification of the training set consisted of G2 (blue) and G1 (red) from the GC168 cohort. Receiver operating curve (ROC) for this dataset. The area under the receiver operating curve (AUC) is 90.0% and 95% confidence interval (CI) is 0.82-0.99. SBS18, SBS44, MSI status, Lauren types and clinical stages for model analysis extracting from the GC168 cohort. Classification of the test set consisted of G2 (blue) and G1 (red) from the ICGC cohort. Receiver operating curve (ROC) for this dataset. The area under the receiver operating curve (AUC) is 74% and 95% confidence interval (CI) is 0.56–0.93. SBS18, SBS44, MSI status, Lauren types and clinical stages for model analysis extracting from the ICGC cohort. G2 refers to the samples with survival of less than 5 years but died, and G1 refers to the samples with a survival of more than 5 years. **(B)** Mean Decrease Accuracy and Mean Decrease Gini display of these 22 biomarkers in random forest.
